# The Regents of New York and the D.D.S. Degree

**Published:** 1902-07

**Authors:** 


### Editorial.

### THE REGENTS OF NEW YORK AND THE D.D.S. DEGREE.

It may not be generally known that for more than a year past there has been a strong influence brought to bear on the Board of Regents of New York to confer the title D.D.S. upon a certain number of estimable gentlemen in the State, and some beyond its borders, who now hold what is known as the M.D.S., an honorary title given by authority of the State some years ago to individuals who had honorably sustained the reputation of dentistry.

There was not at the time serious objection made to this, as it meant no more than any other honorary title and in no way conflicted with the educational degree given by the chartered dental colleges of the country.

A year ago an attempt was made by the Dental Society of the State of New York to induce the Regents to change the M.D.S. to that of D.D.S. It was, doubtless, imagined that the voice of this august body would at once claim attention and the matter would be settled without contention; and further, that in a short time a number of persons of excellent personal reputation would stand upon the same professional equality with college graduates, writing D.D.S. after their names, with all the honor thereunto attached. They probably forgot, in their elation, that some members of the State Society had laboriously taken the curricula of the dental schools and had earned the degree by continuous labor and at serious cost financialy, and were not disposed to see the title given away at the capital of the State, and that by the highest legalized body governing and guarding its educational interests. They felt that if the Regents recognized this demand, the fair name of the State of New York would be smirched and the influence of the board, as an educational power, be gone forever. It was also felt that if this be attempted under the sanction of legislative authority, it would place the State in the odious position of granting unearned degrees.

At the recent meeting of the Dental Society of New York, May 14 and 15, 1902, the subject was again brought before that body and was warmly discussed. The Association of Faculties and dental colleges in general very appropriately received severe castigation. It would, of course, have been expected that those who favored securing the D.D.S. through a written order from the Regents would have no use for dental colleges. The manufacturers of diplomas have no need for schools, but have a decided claim upon the engravers and lithographic press.

The matter is again before the Regents of New York and will probably be decided in July, if not postponed. This, it is hoped, will be done, in order to secure an opinion from the National Associations representing dentistry, which will not meet until the last week in July.

It may be assumed that these national bodies have nothing to do with this question, that it is one simply of local interest. The answer to this would be that New York does not own the degree of D.D.S. (Doctor of Dental Surgery). It has become national in character and international in reputation, and when a certain body, to its discredit, tries to smirch this degree and lower its value, it is not only the right but the duty of all dental college graduates, the world over, to rise up and demand that this deed shall not be done. New York as a State, majestic as that is, is nothing, but the principle involved and sought to be violated is everything.

At the meeting of the National Association of Dental Faculties, held at Milwaukee, August 2 to 6, 1901, the following resolutions were passed:

WHEREAS, The New York Legislature, in a bill signed March 28, 1901, gave to the Board of Regents authority to exchange the M.D.S. degree for the D.D.S. degree, the former, M.D.S., is strictly a local degree conferred by the Dental Society of this State; and

WHEREAS, The said Board of Regents at its annual meeting, July 1, 1901, declined to exercise the power conferred, and referred the subject to the New York State Dental Society for a more definite report on the matter; therefore, be it

*Resolved,* That the thanks of the National Association of Dental Faculties be tendered the Board of Regents of the State of New York for its action postponing a final decision, thus giving opportunity for protests against the proposed action.

*Resolved,* That the National Association of Dental Faculties respectfully protest against any attempt to undo the work of this Association, which for many years has had in force a law which does not permit the granting of the degree of Doctor of Dental Surgery (D.D.S.) except at the close of a course of study in some accredited school and on proper examination.

*Resolved,* That we further earnestly and respectfully ask that the authority thus given the Regents be rescinded.

*Resolved,* That a copy of these resolutions, signed by the President and Secretary, be sent to the Regents' office at Albany, N. Y., and also to the Secretary of the New York State Society, W. A. White, Phelps, N. Y.

Further action by the Board of Regents was postponed until the dental profession of New York could be heard from. This, it is presumed, meant until the State Society could again revise its decision. This was done, as before stated, last May, and the resolution favoring the change of title was adopted by an overwhelming majority. It was expected that this would settle the question and that those who desired to maintain the integrity of the degree would quietly settle down and await results. Fortunately all men are not "cravens when the storm gathers," and there has been a strong effort made to counteract this decision of the State Society. Whether this will be any more successful remains for time to determine.

The history of the origin of the D.D.S. degree has yet to be written. It was the outgrowth through a declared and positive opposition of the medical fraternity to anything dental. This piece of stupid arrogance on the part of medical men forced Harris and his colleagues to originate a distinct title never before known, that of Doctor of Dental Surgery, or abbreviated D.D.S. This degree was made legal by charter rights conferred by the State of Maryland. It was born in difficulty and nursed into life through long and arduous effort by the dental colleges of the country, and no attempt was made in the United States to substitute another until Harvard University adopted the degree D.M.D. (Dentariæ Medicinæ Doctor) as being more appropriate. Time has intensified the love for the D.D.S., and the graduates of our dental colleges, now performing professional work in all lands, will not willingly stand by and see their hardly earned title dragged in the mire, and that by one of the most rigid educational bodies in America,—the Board of Regents of the State of New York.

The effect of this order of the Regents would be to practically confer an honorary degree upon the worthy gentlemen now holding the M.D.S. title. Honorary degrees have been long since abolished in professional schools, and in order that subordinate dental colleges might not make the mistake of conferring this degree the Faculties passed, some years since, the following resolution, as a part of the organic law of the organization. "No college connected with the Association shall confer any degree as honorary which is usually granted in due course of study and examination. All former rules on this subject are hereby repealed." Frequent applications have been made by colleges connected therewith to be given the privilege of conferring this degree upon distinguished men in this country and Europe, but they have invariably been refused. The Faculties has no power over the Board of Regents, but if it should give the D.D.S. to the gentlemen demanding it, there will be manifest a power behind that of the college organization, and, greater than it, the dental profession, that will assuredly hold these easily secured honors in supreme contempt. In this day and generation men and women are judged by their ability, and the degree legally held is simply a standing certificate that the holder thereof has laid well the foundation of knowledge and has been recognized by his peers. The man without the degree is equally recognized by the great body of his colleagues, and has no need to add to his name any letters, or to cover his breast with medals of honor, for his reputation is known of men. The writer has small sympathy with this craze for titles. When the degree of LL.D. is given to all sorts of men and for all sorts of service, in which learning occupies but a small part, the self-respecting individual may well hesitate in accepting empty honors, and may ask themselves, Better be plain John Smith with his mental power than Richard Roe with his aggregations of possible stupidities and sham letters.

If the majority of the members of the Dental Society of the State of New York are in favor of conferring honorary degrees, they may be assured that the most intelligent of the dental profession will hold them to a strict accountability, for by this they have forced an entering wedge that will eventually sever into its original elements the growth of fifty years of educational labor. The principles which enter into the life of professions are well grounded and understood of all men, and are the result of the combined experience of generations. To tamper with these means a retrograde and a loss which may not be overcome in a century of work. May the Regents of New York carefully consider that upon their action will depend not the mere changing a title, however small or great that may be, but it may be the means of destroying the confidence in the stability and trustworthiness of men who have had in charge the educational work of the country.

